# FBG Interrogation Method with High Resolution and Response Speed Based on a Reflective-Matched FBG Scheme

**DOI:** 10.3390/s150716516

**Published:** 2015-07-08

**Authors:** Jiwen Cui, Yang Hu, Kunpeng Feng, Junying Li, Jiubin Tan

**Affiliations:** Institute of Ultra-precision Optoelectronic Instrument Engineering, Science Park, Harbin Institute of Technology, No. 2 Yikuang Street, Nangang District, Harbin 150080, China; E-Mails: hithy@hit.edu.cn (Y.H.); aifenglin@gmail.com (K.F.); duckhome@163.com (J.L.); jbtan@hit.edu.cn (J.T.)

**Keywords:** FBG interrogation method, reflective-matched FBG, high wavelength resolution, high response speed

## Abstract

In this paper, a high resolution and response speed interrogation method based on a reflective-matched Fiber Bragg Grating (FBG) scheme is investigated in detail. The nonlinear problem of the reflective-matched FBG sensing interrogation scheme is solved by establishing and optimizing the mathematical model. A mechanical adjustment to optimize the interrogation method by tuning the central wavelength of the reference FBG to improve the stability and anti-temperature perturbation performance is investigated. To satisfy the measurement requirements of optical and electric signal processing, a well- designed acquisition circuit board is prepared, and experiments on the performance of the interrogation method are carried out. The experimental results indicate that the optical power resolution of the acquisition circuit border is better than 8 pW, and the stability of the interrogation method with the mechanical adjustment can reach 0.06%. Moreover, the nonlinearity of the interrogation method is 3.3% in the measurable range of 60 pm; the influence of temperature is significantly reduced to 9.5%; the wavelength resolution and response speed can achieve values of 0.3 pm and 500 kHz, respectively.

## 1. Introduction

Thanks to its immunity to electromagnetic interference, high accuracy and long term stability the FBG technique has been wildly used in the fields of communication [1], dimensional metrology [2], building health monitoring [3,4], ultrasonic wave and vibration measurement [5], the petrochemical industry [6], and other harsh and remote environments. The sensing principle of FBG sensors involves detecting the central wavelength variation. Nevertheless, conventional spectrum interrogation methods utilizing an optical spectrum analyzer (OSA) with low resolution and response speed cannot satisfy the requirements of accurately detecting the small and dynamic variations of the FBG’s central wavelength. Therefore, it is of significance to find a FBG interrogation method with high resolution and high response speed.

In recent years, much work has been done on this particular aspect. For example, in 2014 Potts, *et al.* [7] fabricated tapered hollow Bragg waveguides coupled to an image sensor to extract spectral shifts on the basis of the wavelength dependence of the light radiated at mode cutoff. Though the operational range of the method could be hundreds of nanometers, a resolution of ~10 pm by employing a peak detection algorithm and an insufficient detection response limited by an image sensor could not satisfy the requirements of high accuracy measurement. Ma, *et al.* [8] demonstrated in 2013 a FBG sensor interrogation method based on fiber a Fabry–Perot tunable filter (FFP-TF) and Fabry-Perot ITU filter (FPIF), and the resolution achieved was better than 2 pm. However, the fineness of the FFP-TF should be increased to improve the resolution which will induce larger insertion losses and reduce the signal-noise-ratio (SNR). Later, to address the higher resolution issue, Yang, *et al.* [9] described in 2014 a FBG interrogation method which used a Fabry-Perot etalon as a dynamical calibration and wavelength reference. They achieved a resolution of 0.23 pm with the method of segmentation interrogation using the ASE spectral characteristics. Nevertheless, the segmentation process limits the speed of this interrogation method. In 2011 Kim and Song [10] proposed a linear FBG interrogation method utilizing a tunable wavelength laser and a volume phase grating spectrometer. Though they got a linear response *versus* the Bragg wavelength shift and a much higher SNR compared with the conventional spectrometer interrogation method with much dimmer broadband light sources, a low interrogation speed still limits its practical applications.

Among the methods proposed above, the resolution and response speed are the key issues of the FBG interrogation method, especially in the field of ultrasonic wave and vibration measurement. To maintain a high response speed and simultaneously achieve high resolution are the current research focus in the field of FBG interrogation methods.

In this article, a FBG interrogation method based on the reflective-matched FBG scheme by detecting the optical power ratio is proposed [11]. The nonlinear problem of conventional reflective-matched FBG sensing interrogation schemes is solved by establishing and optimizing the mathematical model of the interrogation scheme to get the approximately linear and most sensitive matched conditions. Moreover, calculating the power ratio can suppress the optical power fluctuation compared with detecting the optical power directly. As a result, the achievable resolution and response speed of our interrogation method are sub-picometer, and several hundreds of kHz, respectively.

## 2. Principle of the Proposed FBG Interrogation Method

### 2.1. Principle of the Interrogation Method

**Figure 1:**
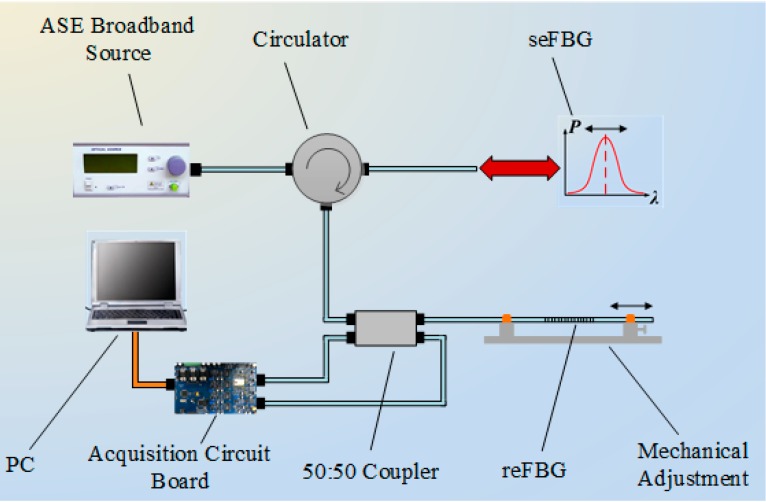
Schematic diagram of the FBG interrogation method based on a reflective-matched FBG scheme.

Figure 1 shows the schematic diagram of the FBG interrogation method based on reflective-matched FBG scheme. A light emitted from amplified spontaneous emission (ASE) broadband source enters the sensing FBG (seFBG) through a circulator, and the reflection spectrum of seFBG then propagates back through the circulator again to the 50:50 coupler, where the reflected signal is divided into two parts of equal power. A part of the light is received by the photodiode of channel 1 on the acquisition circuit board, and the other part is filtered and reflected again by the reference FBG (reFBG). The reflection spectrum of the reFBG is received by the photodiode of channel 2 on the same acquisition circuit board.

**Figure 2:**
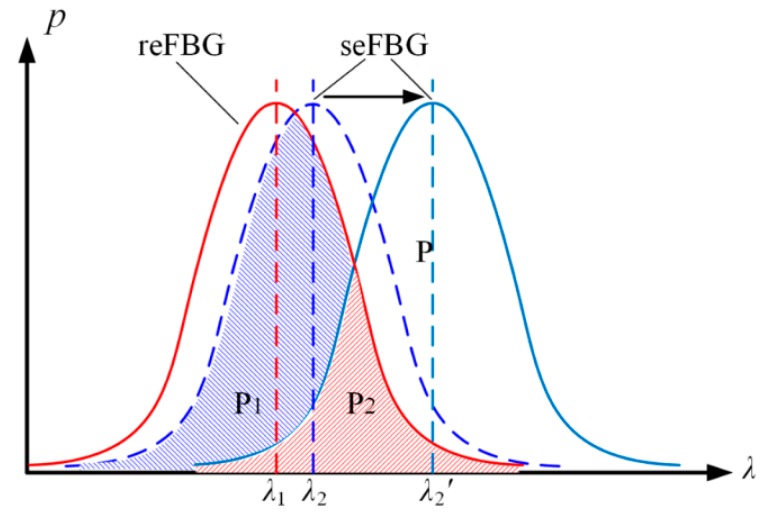
Principle of the proposed FBG interrogation method: P is the reflection spectrum power of seFBG; $P_{1}+P_{2}$ is the initial overlapping reflection spectrum power of the seFBG and the reFBG; $P_{2}$ is the overlapping reflection spectrum power of the seFBG and the reFBG after the influence of the external physical parameter.

The proposed FBG interrogation method based on a reflective-matched FBG sensing interrogation scheme demodulates the central wavelength shift of the seFBG on the basis of the ratio of the overlapping reflection spectrum power of the seFBG and the reFBG to the reflection spectrum power of the seFBG. As shown in Figure 2, the central wavelength of the reFBG is $\lambda_{1}$, and the initial central wavelength of the seFBG is λ_*2*_. Meanwhile, the initial ratio of their overlapping power to the reflection spectrum power of the seFBG is $(P_{1}+P_{2})/P$. Due to the effect of the external physical parameter, the central wavelength of the seFBG shifts to $\lambda_{2}^{'}$ and the power ratio becomes $P_{2}/P$. Moreover, calculating the power ratio can suppress the optical power fluctuation compared with detecting the optical power directly.

The FBG’s reflection spectrum can be expressed as [12]:

(1) $$I(\lambda )=\exp\left[-4\ln2{\left(\frac{\lambda -\lambda_{B}}{\Delta \lambda }\right)}^{2}\right]$$

where ${\text{λ}}_{B}$ is the central wavelength of the FBG; Δλ is the full width at half maximum (FWHM) of the FBG’s reflection spectrum.

Hence, the power of the FBG’s reflection spectrum can be expressed as:

(2) $$\begin{array}{ll} I & =\int_{-\infty }^{+\infty }\exp\left[-4\ln2{\left(\frac{\lambda -\lambda_{B}}{\Delta \lambda }\right)}^{2}\right]d\lambda \\ \; & =\Delta \lambda \sqrt{\frac{\pi }{4\ln2}} \end{array}$$

According to Equations (1) and (2) above, the overlapping power of the seFBG and reFBG’s reflection spectrum can be expressed as:

(3) $$\begin{array}{ll} P(\lambda_{1},\lambda_{2}) & =\int_{-\infty }^{+\infty }\exp\left[-4\ln2{\left(\frac{\lambda -\lambda_{1}}{\Delta \lambda_{1}}\right)}^{2}\right]\exp\left[-4\ln2{\left(\frac{\lambda -\lambda_{2}}{\Delta \lambda_{2}}\right)}^{2}\right]d\lambda \\ \; & =\frac{\exp\left[-4\ln2\frac{{\left(\lambda_{1}-\lambda_{2}\right)}^{2}}{\Delta \lambda_{1}^{2}+\Delta \lambda_{2}^{2}}\right]}{\sqrt{\frac{4\ln2}{\pi }\left(\frac{1}{\Delta \lambda_{1}^{2}}+\frac{1}{\Delta \lambda_{2}^{2}}\right)}} \end{array}$$

where ${\text{λ}}_{1}$ and ${\text{λ}}_{2}$ are the central wavelength of the seFBG and reFBG, respectively; $\Delta {\text{λ}}_{1}$ and $\Delta {\text{λ}}_{2}$ are the FWHM of spectra of the seFBG and reFBG, respectively. Therefore, the power ratio of the optical signals received by the acquisition circuit board can be expressed as:

(4) $$R(\lambda_{1},\lambda_{2})=\frac{P(\lambda_{1},\lambda_{2})}{I}=\frac{\exp\left[-4\ln2\frac{{\left(\lambda_{1}-\lambda_{2}\right)}^{2}}{\Delta \lambda_{1}^{2}+\Delta \lambda_{2}^{2}}\right]}{\Delta \lambda_{1}\sqrt{\left(\frac{1}{\Delta \lambda_{1}^{2}}+\frac{1}{\Delta \lambda_{2}^{2}}\right)}}$$

In experiments, reFBGs and seFBGs fabricated under the same conditions are used, and the FWHM of the seFBG and reFBG are approximately equal, $\Delta \lambda_{1}\approx \Delta \lambda_{2}=\Delta \lambda$. Hence Equation (4) can be simplified as:

(5) $$R(\lambda_{1},\lambda_{2})=\frac{P(\lambda_{1},\lambda_{2})}{I}=\frac{\exp\left[-2\ln2\frac{{\left(\lambda_{1}-\lambda_{2}\right)}^{2}}{\Delta \lambda^{2}}\right]}{\sqrt{2}}\;$$

**Figure 3:**
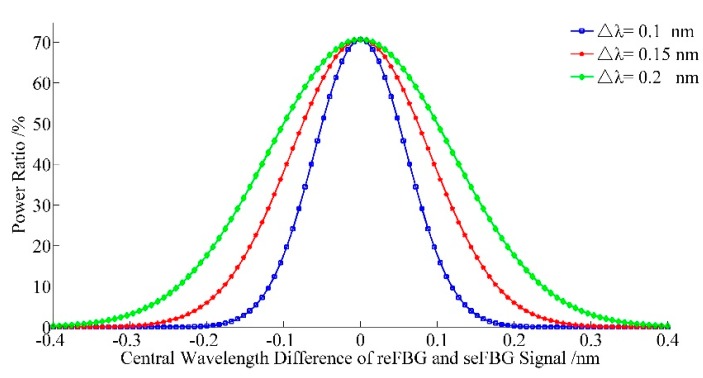
Relation between the power ratio and the central wavelength difference between the reFBG and seFBG while the FWHM of the seFBG and reFBG are equal.

According to the result of Equation (5), the relation between the power ratio and the central wavelength difference of the reFBG and seFBG can be established as shown in Figure 3. It indicates that the FWHM of the reFBG has an effect on the sensitivity of the interrogation method, and the sensitivity can be improved by configuring reFBG with a narrow FWHM. Considering that the reFBG is unaffected by the measured physical parameter, the shift of the central wavelength difference between the reFBG and seFBG can be simplistically regarded as the central wavelength shift of the seFBG. Therefore, the relation between the power ratio and the central wavelength shift of the seFBG is nonlinear, but there is an approximately linear and most sensitive section. Then the existence of this section is proved, and the condition to optimize the interrogation method working in its approximately linear and most sensitive mode is established.

The first order differential of the power ratio $\nabla R(\lambda_{1},\lambda_{2})$ is the gradient of the power ratio induced by an external influence which characterizes the sensitivity of the interrogation method; the second order differential of power ratio $\nabla^{2}R(\lambda_{1},\lambda_{2})$ is the gradient of the power ratio’s variation, and the interrogation method works in its optimized mode when $\nabla R(\lambda_{1},\lambda_{2})$ reaches its maximum and $\nabla^{2}R(\lambda_{1},\lambda_{2})$ is zero. To obtain the relation between the central wavelength of the seFBG and reFBG, the following calculations are implemented. The first order differential of the power ratio is:

(6) $$\frac{\partial R(\lambda_{1},\lambda_{2})}{\partial \lambda_{2}}=-8\ln2\frac{(\lambda_{1}-\lambda_{2})\exp\left[-2\ln2\frac{{\left(\lambda_{1}-\lambda_{2}\right)}^{2}}{\Delta \lambda^{2}}\right]}{2\sqrt{2}\Delta \lambda^{2}}$$

**Figure 4:**
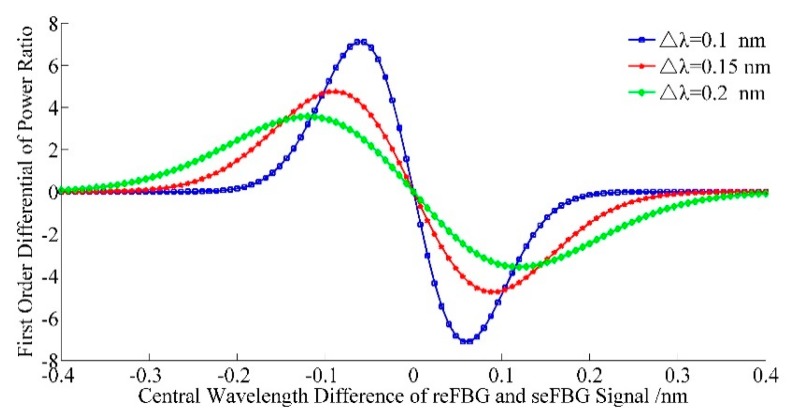
Relation between the first order differential of the power ratio and central wavelength difference between the reFBG and seFBG.

As shown in Figure 4, the first order differential reaches its extrema twice, corresponding to two optimized modes of the interrogation method.

The second order differential of the power ratio is:

(7) $$\frac{\partial^{2}R(\lambda_{1},\lambda_{2})}{\partial \lambda_{2}^{2}}=8\ln2\frac{\left[8\ln2{\left(\lambda_{1}-\lambda_{2}\right)}^{2}-2\Delta \lambda^{2}\right]\exp\left[-2\ln2\frac{{\left(\lambda_{1}-\lambda_{2}\right)}^{2}}{\Delta \lambda^{2}}\right]}{2\sqrt{2}\Delta \lambda^{2}}$$

Let $\frac{\partial^{2}R(\lambda_{1},\lambda_{2})}{\partial \lambda_{2}^{2}}=0$, the relation between the central wavelengths of the seFBG and reFBG to optimize the method working in its optimized mode can be expressed as:

(8) $$\left|\lambda_{1}-\lambda_{2}\right|=\frac{\Delta \lambda }{1.6651}$$

Therefore, the optimal initial matching condition to optimize the interrogation method working in an approximately linear and most sensitive mode is established when the initial central wavelengths of the seFBG and reFBG satisfy Equation (8). The central wavelength of the reFBG is known and the constant during the measurement process and the central wavelength variation of the seFBG can thus be demodulated.

It is shown in Equation (5) and Figure 3 that the nonlinearity of interrogation method is determined by the measurable range. Assuming that the FWHM of the seFBG and reFBG are 0.2 nm, a measurable range of the central wavelength variation of the seFBG with a nonlinearity of less than 1% can reach ±43 pm, and a measuring range of the central wavelength variation of the seFBG with a nonlinearity of less than 5% can reach ±85 pm. It is sufficient for the application of the FBG sensors.

**Figure 5:**
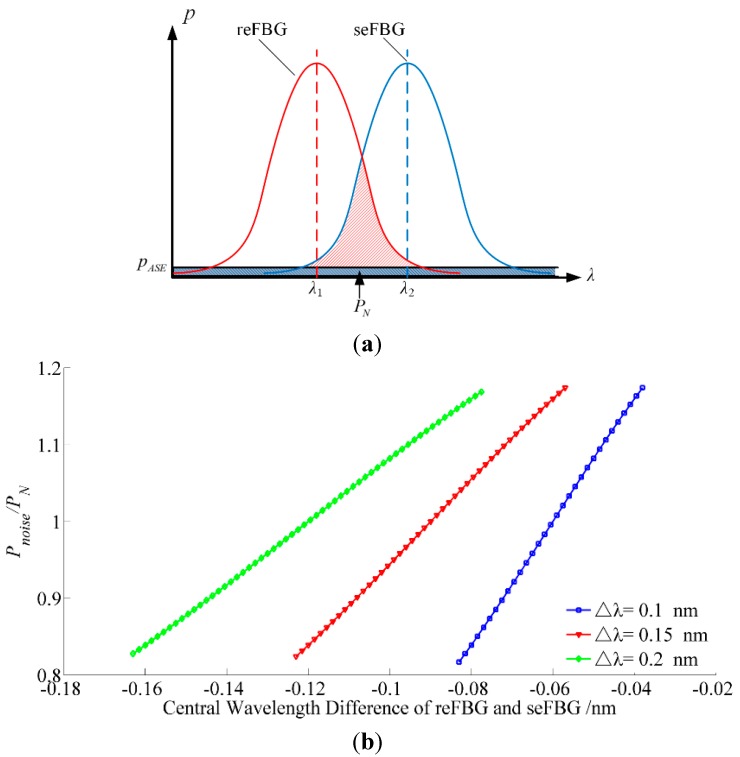
Analysis of the noise power introduced by ASE broadband source: (**a**) Principle of the noise level analysis; (**b**) the relation between $P_{noise}/P_{N}$ and central wavelength difference in the most sensitive section with the nonlinearity of 1%.

The noise level varies with the central wavelength of the reFBG and seFBG and it is necessary to investigate its influence and the performance extremes of the interrogation method. The noise of the ASE broadband source can be assumed to be a band-limited white noise with a noise power spectrum density (NPSD) of $p_{ASE}$. Meanwhile, the noise power within the received optical signal of the overlapping reflection spectrum, is $P_{noise}(\lambda_{1},\lambda_{2},\Delta \lambda )$ and it reaches $P_{N}$ under the optimal initial matching condition as shown in Figure 5a. The relation between $P_{noise}(\lambda_{1},\lambda_{2},\Delta \lambda )/P_{N}$ and the central wavelength difference with a nonlinearity of less than 1% is established in Figure 5b. It can be seen that the noise level increases with the overlapping reflection spectrum of the reFBG and seFBG and it reaches the maximum at the edge of the linear working range with a minimum central wavelength difference of the reFBG and seFBG that means the interrogation method works at its extreme performance, so the interrogation method is tested under conditions close to the edge of the linear working range, including the minimum detectable optical power.

### 2.2. Analysis of the Relation between the FWHM and the Optimal Initial Matching Condition

**Figure 6:**
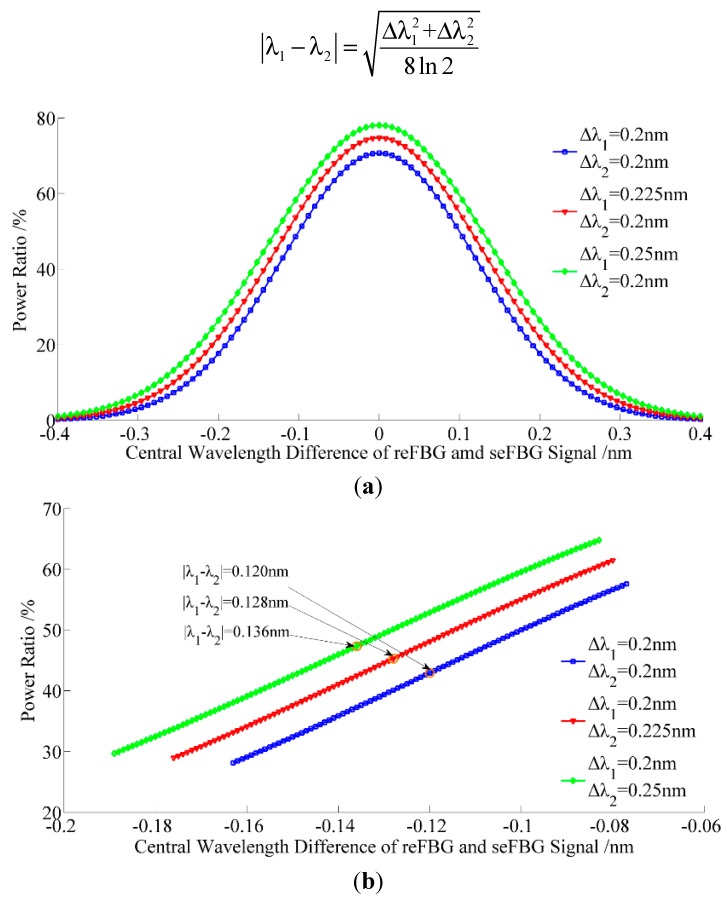
Relation between the power ratio and the central wavelength difference between the reFBG and seFBG while the FWHM of the seFBG and reFBG are not equal: (**a**) full scale (**b**) most sensitive section with the nonlinearity of 1%.

The seFBG and reFBG’s spectra are usually different, even they have same grating parameters. The main difference between the seFBG and reFBG is their FWHM as a result of the fabrication errors or the spectral broadening introduced by the high frequency vibration of the seFBG in frequency response experiments. Figure 6a shows the relation between the central wavelength difference and the power ratio with different FWHMs of the seFBG and reFBGs’ spectra. It can be seen that the approximately linear section still exists without losing sensitivity, but the initial power ratio and measurable range increases and the optimal initial matching condition changes according to Figure 5b. The optimal initial matching condition is practical on the condition that the FWHMs of the seFBG and reFBG is not equal, and it can be driven from the steps of Equations (6) and (7) and given in a universal expression as:

(9) $$\left|\lambda_{1}-\lambda_{2}\right|=\sqrt{\frac{\Delta \lambda_{1}^{2}\text{+}\Delta \lambda_{2}^{2}}{8\ln2}}$$

The spectral broadening of the seFBG can be observed in the frequency response experiment. It can be seen from Figure 6b that the spectral broadening causes the variations of optimal initial matching condition and the initial power ratio which have effects on the initial signal. However, the frequency response of the interrogation is not influenced by these variations and the relation between the power ratio and the central wavelength shift of the seFBG can be calibrated before the experiments using a vibration with a known peak-to-peak strain.

### 2.3. Principle of the Mechanical Adjustment to Satisfy the Optimal Initial Matching Condition

As noted above, to optimize the method working in its optimized mode, a central wavelength adjustment means of the reFBG is applied. First, we choose a reFBG whose initial central wavelength is similar to and slightly smaller than the central wavelength of the seFBG. Then, we utilize the mechanical adjustment to stretch the reFBG and adjust its central wavelength to the optimal initial matching condition. The central wavelength of the reFBG depends on the effective index of refraction of the core $n_{eff}$ and the period of the grating through the resonance condition $\lambda_{2}\text{ = }2n_{eff}\Lambda$. The effective index of refraction, as well as the period, will be affected by the axial stretching strain. The variation of the central wavelength $d{\text{λ}}_{2}$ due to the axial strain changes Δε is given by:

(10) $$d\lambda_{2}=\lambda_{2}(1-p)\Delta \varepsilon$$

where *p* is the photoelastic coefficient of the fiber, given by:

(11) $$p=\frac{{n_{eff}}^{2}}{2}\left[p_{12}-v(p_{11}-p_{12})\right]$$

where $p_{11}$ and $p_{12}$ are the components of the fiber-optic strain tensor and *v* is Poisson’s ratio, and *p* = 0.22 for a typical optical fiber. Accordingly, the central wavelength of the reFBG linearly varies with the axial strain applied by the mechanical adjustment.

### 2.4. Principle of the Anti-temperature Perturbation of the Interrogation Method

The temperature-sensitivity of FBG sensors influences their accuracy and limits their application for precise measurements. The cross-sensitivity of strain and temperature is the key problem of the FBG sensors. Several approaches have been implemented to compensate or extract the temperature variation, such as FBG sensors with a sandglass-shape [13], FBG sensors with an additive micro-scale bi-material coating [14], FBG sensors using a Fabry-Perot laser diode and a dual-stage FBG optical demultiplexer [15]. However, the proposed interrogation method could reduce the temperature sensitivity of the FBGs without any extra approaches by employing dual mutually referenced FBGs with a same thermo-optic coefficient and thermal expansion in a uniform environment.

In conventional interrogation methods without a reference FBG, the central wavelength variation of the seFBG $\delta \lambda_{1}$ for a temperature change ΔT can be written as:

(12) $$\delta \lambda_{1}=\lambda_{1}(\alpha_{B}+\xi )\Delta T$$

where, $\alpha_{B}=(1/\Lambda )(\partial \Lambda /\partial T)$ is the thermal expansion coefficient of the fiber (0.55 × $10^{-6}$/°C for a germanium doped silica-core fiber); ξ=(1/n)(∂n/∂T) is the thermos-optic coefficient (8.6× $10^{-6}$/°C for a germanium doped silica-core fiber).

The reFBG adjusted by the mechanical adjustment in the proposed interrogation method compensates the drift caused by the direct effect of temperature variation during the measurement. Meanwhile, the error resulting from the axial strain changes as a result of the different thermal expansion of the silica-core fiber and the mechanical adjustment was introduced. Consequently, the central wavelength variation of the reFBG $\delta \lambda_{2}$ due to the temperature change ΔT can be written as:

(13) $$\delta \lambda_{2}=\lambda_{2}(\alpha_{B}+\xi )\Delta T+\lambda_{2}(1-p)\alpha_{m}L\Delta T$$

where, $\alpha_{m}$ is the thermal expansion coefficient of the mechanical adjustment (23.6 × $10^{-6}$/°C for aluminium alloy); *L* is the fiber length exposed between the two fixtures of the mechanical adjustment which is 3 cm long for the experiment. Considering that the central wavelength of the seFBG and reFBG are close, and the proposed interrogation method demodulates the central wavelength of seFBG according to the shift of the central wavelength difference between the reFBG and seFBG, the effect of temperature on the proposed interrogation method compared with the conventional interrogation method can be expressed as:

(14) $$u=\left|\frac{\Delta \lambda_{2}-\Delta \lambda_{1}}{\Delta \lambda_{1}}\right|\times 100\%\approx \left|\frac{(1-p)\alpha_{m}L}{\alpha_{B}+\xi }\right|\times 100\%=6.04\%$$

Therefore, the proposed interrogation method can effectively reduce the influence of the temperature drift.

## 3. Experiments and Results

In order to demonstrate that the proposed FBG interrogation method can effectively improve the resolution and response speed, experiments on the FBG interrogation method based on reflective-matched FBG scheme are conducted. In this section, an acquisition circuit board is designed and manufactured to process optical and electric signals, and then the stability, optical power resolution, nonlinearity, wavelength resolution, frequency response and anti-temperature performance of the proposed FBG interrogation method are verified experimentally.

### 3.1. Design of the Acquisition Circuit Board

**Figure 7:**
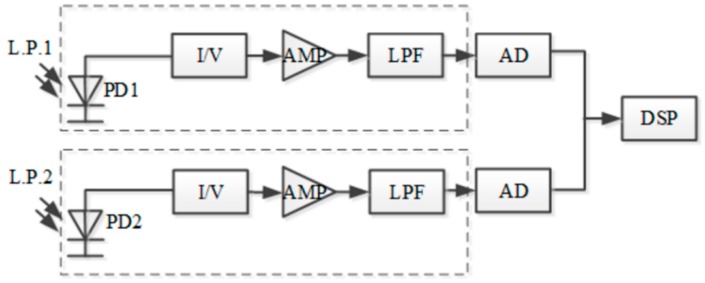
Module design of the acquisition circuit board.

Modules design of the acquisition circuit board is shown in Figure 7. In order to reduce the effects of dark current in weak signal detection, two FGA01FC photodiodes with a bandwidth of 1 GHz operating in photovoltaic mode are used to detect the reflection spectrum power.

Due to the low-current-noise demand of the front-end weak current amplification, an AD795 FET amplifier with a maximum current noise of 0.6 fA/√Hz at 1 kHz is used as a I/V converter. Meanwhile, to reduce the voltage noise of the continued voltage amplification, two OP37 units with a low input voltage-noise of maximum 11 nV/√Hz at 10 kHz are employed to achieve a two-stage amplification. In order to prevent the sampling value oscillation and filter the high frequency noise in the circuit, a fourth order Butterworth lowpass filter is designed and applied between the amplifier section and the A/D converter.

A two-channel simultaneous high-speed sampling 16-bit A/D converter samples the voltage signals converted from the optical power signals and sends the digital signals to a DSP controller. The DSP controller processes the digital signals to achieve the power ratio of the two photodiodes and transmits it to a PC.

**Figure 8:**
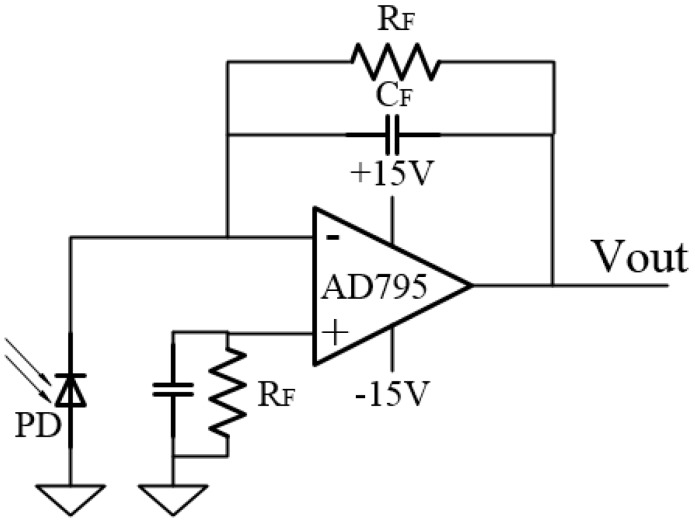
Schematic diagram of the I/V converter.

The feedback resistor and the input capacitance produce a pole in the I/V converter’s loop transmission that can result in peaking and instability, so it is necessary to add a compensation capacitor *C_F_* to create a zero point in the loop transmission that could compensate the effect of the pole and reduce the signal bandwidth as shown in Figure 8. The stable bandwidth attainable with the preamp is a function of the gain bandwidth product of the amplifier (1.6 MHz), *R_F_*, and the total capacitance at the amplifier’s summing junction, *C_IN_*. For this circuit, the FGA01FC photodiode has a maximum capacitance of *C_D_* = 2 pF. The AD795 common-mode input capacitance is *C_M_* = 2.2 pF, and the differential-mode input capacitance is *C_D_* = 2 pF. Therefore, the total input capacitance is C_IN_ = 6.2 pF. The signal bandwidth resulting in a 45° phase margin, *f*_*(*45*)*_, is defined by:

(15) $$f_{\left(45\right)}=\sqrt{\frac{f_{CR}}{2\pi \times R_{F}\times C_{IN}}}=\sqrt{\frac{1.6\text{ MHz}}{2\pi \times 100\text{ kΩ}\times 6.2\text{ pF}}}=0.64\;\mathrm{MHz}$$

The design bandwidth is 500 kHz, which can meet the requirements according to Equation (15). The value of the *C_F_* with the selected value of *R_F_* is:

(16) $$C_{F}=\frac{1}{2\pi R_{F}f_{2}}=\frac{1}{2\pi \times 100\text{ kΩ}\times 500k}=3.2\text{ pF}$$

To determine whether a compensation capacitance of 5.3 pF is enough to stabilize the system, the minimum value of the *C_F_* needed to obtain a 45° phase margin is calculated and can be expressed as:

(17) $$C_{F}=\sqrt{\frac{C_{IN}}{2\pi R_{F}f_{CR}}}=\sqrt{\frac{6.2\text{ pF}}{2\pi \times 100\text{ kΩ}\times 1.6\text{ MHz}}}=2.5\text{ pF}$$

The system is stable because the larger compensation capacitance of 3.2 pF is larger than 2.5 pF and it increases the amount of phase margin. The detection frequency can be even higher by designing an I/V converter circuit with a larger gain bandwidth amplifier according to Equations (15)–(17), and using an A/D converter with a higher sampling frequency.

### 3.2. Experiments on the Performance of the Proposed FBG Interrogation Method

#### 3.2.1. Mechanical Adjustment Experiments to Satisfy the Optimal Initial Matching Condition

The initial central wavelength of the seFBG and reFBG in the experiment are 1549.256 nm and 1539.148 nm, respectively. The FWHMs of both FBGs are 0.2 nm. The optimal initial matching condition of $|\lambda_{1}-\lambda_{2}|=0.120\text{ nm}$ to optimize the system working in its optimized mode can be calculated according to Equation (8). The central wavelength of the reFBG is adjusted to 1549.376 nm through stretching the reFBG with the mechanical adjustment.

The reFBG’s central wavelength is adjusted to achieve the optimal initial matching condition and the central wavelength should remain stable after the adjustment, so the stability of the reFBG’s central wavelength on the mechanical adjustment is tested.

**Figure 9:**
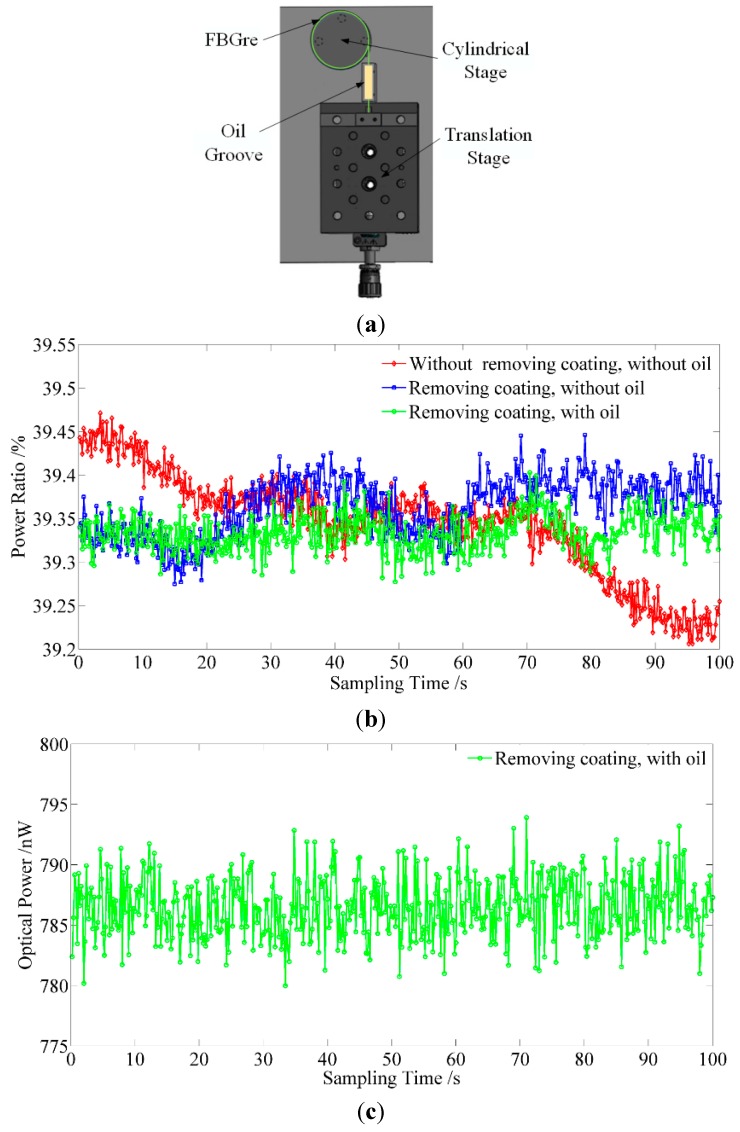
Experiment setup and result of the mechanical adjustment: (**a**) schematic diagram of the mechanical adjustment; (**b**) experimental results of the stability of the power ratio with reFBG stretched by the mechanical adjustment; (**c**) experimental results of the stability of the overlapping optical power.

As shown in Figure 9a, the mechanical adjustment system to satisfy the optimal initial matching condition consists of a cylindrical stage, oil groove, and translation stage. The dimensions of the mechanical adjustment system are 7 cm × 5 cm × 1.5 cm, which is small enough to ensure the reFBG and seFBG are close in the environment of a clean room with the same temperature gradient. One end of the fiber comprising the reFBG is twined around the cylindrical stage, and the other end is compacted on the translation stage through the oil groove. A pulling force is applied on the reFBG by adjusting the translation stage.

Figure 9b indicates the experimental results of the stability of the power ratio with reFBG stretched by the mechanical adjustment, and the simultaneous stability of the overlapping reflection spectrum power of the seFBG and the reFBG is shown in Figure 9c. As the main material of coating is acrylate whose tensile strength is about 70 MPa, which it is weaker than the 110 MPa of silicon dioxide (the cladding material), in the experiment, the front end of the translation stage is used to clamp the reFBG. The coating is easily destroyed and separated from the cladding in the adjustment process, which causes stress relief and influences the stability. While the coating is removed before adjustment, the tensile strength of the cladding is high enough to ensure that no destructive deformation happens.

On the other hand, the compressive strength of acrylate is 90 MPa and that of silicon dioxide is 1000 MPa. The pressure applied to the fiber should not be higher than the compressive strength of the material, but high enough to avoid any stress relief. Destructive deformation occurs easily on the coating, and relative slip between the fiber and the translation stage happens, while the pressure that the cladding can bear is ten times higher than that of the coating, so it’s easy to infer that a much smaller stress relief can be guaranteed without coating. Therefore, the coating of the reFBG should be removed before it is clamped on the translation stage. The oil groove the FBG part is wrapped in is used to increase the damping, and can reduce the influence introduced by ambient vibration. In initial testing, the stability cannot meet the requirements because of the stress relief when the coating is destroyed in the adjustment process, so the coating is removed before adjustment, and the stability is improved to 0.06% with a signal fluctuation of 0.152% by immersing the fiber comprising the reFBG to the oil groove to decrease the fiber length exposed to air and diminish the influence of air turbulence. However, the stability of directly detecting the overlapping optical power is 14 nW with a signal fluctuation of 1.783%. It can be concluded that the power ratio detection can further improve the percentage of the signal fluctuation by suppressing the optical power fluctuation.

#### 3.2.2. Experiment on the Optical Power Resolution of the Acquisition Circuit Board

**Figure 10:**
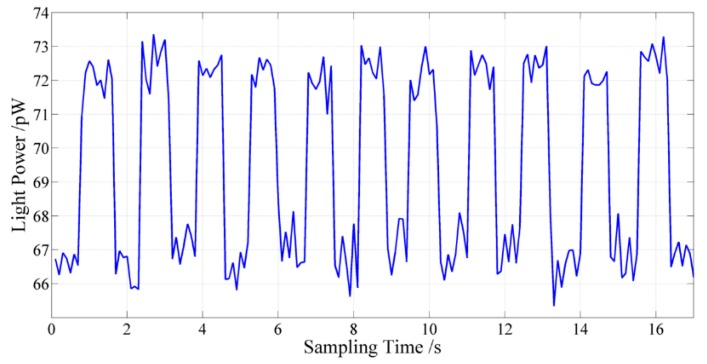
Experimental result of the optical power resolution of the acquisition circuit board.

In consideration of the weak reflection spectrum power and the interrogation scheme based on detecting the optical power, the optical resolution of the acquisition circuit board is very necessary. To meet the needs of weak signal detection as mentioned above, the optical power resolution of the acquisition circuit board is tested. The electro-optical modulator driven by a square wave is used to modulate the optical signal. Then the modulated optical signal is detected by the acquisition circuit board, and the optical power resolution result is achieved by adjusting the amplitude square wave. Figure 10 indicates that the achievable optical power resolution is better than 8 pW.

#### 3.2.3. Experiment on the Linearity of the Proposed Interrogation Method under the Optimal Initial Matching Conditions

The proposed interrogation method linearly transforms the central wavelength variation of the seFBG into the power ratio according to Equation (4) under the optimal initial matching conditions; hence an experiment on the nonlinearity of the interrogation method is conducted.

**Figure 11:**
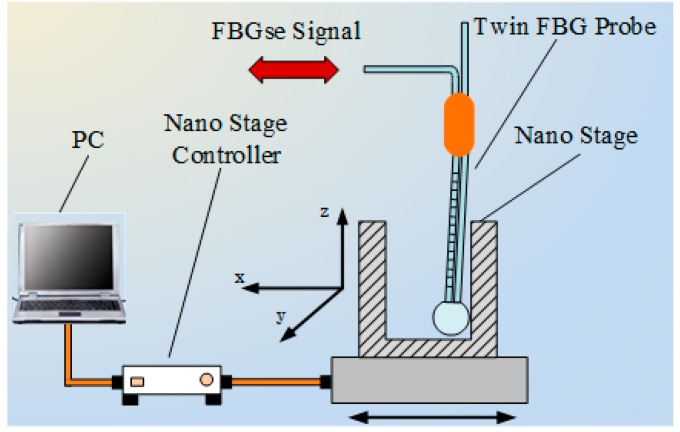
Schematic diagram of seFBG axial displacement signal generator.

**Figure 12:**
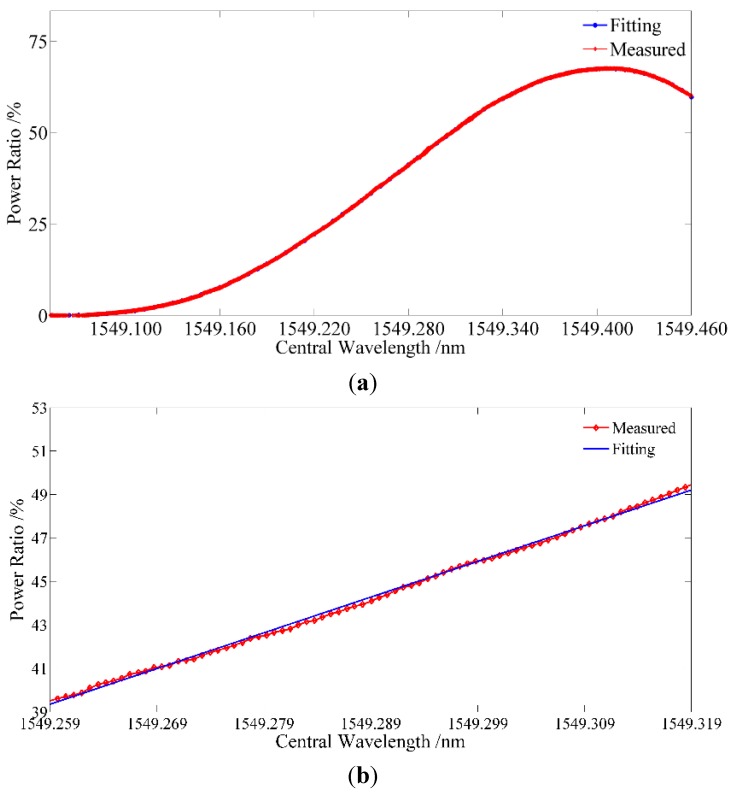
Experimental result of the linearity of the interrogation method: (**a**) the optical power ratio on a large scale; (**b**) the optical power ratio on available measurement scale.

The seFBG signal is generated by a twin FBG probe with a radial touch of a nano stage working in servo mode [2]. The seFBG is bent through the radial contact to get the linear variation of central wavelength as shown in Figure 11. Figure 12a shows the optical power ratio of the overlapping reflection spectrum power of the seFBG and reFBG to the reflection spectrum power of the seFBG on a large scale, while Figure 12b shows the available measurement scale.

The red curve is the actual measured result while the blue curve is the fitting result. It indicates that the nonlinearity of the interrogation method is 3.3% in the measurable range of 60 pm which is basically consistent with the theoretical simulation. Therefore, the proposed interrogation method has a good nonlinearity and thus can be commendably applied to interrogate FBG sensors under the optimal initial matching conditions.

#### 3.2.4. Experiment on the Wavelength Resolution

**Figure 13:**
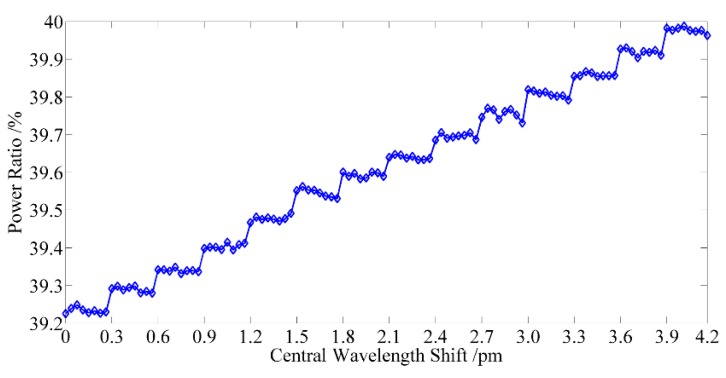
Experimental result of the wavelength resolution of the interrogation method.

To measure the wavelength resolution of the proposed interrogation method which is one of the most important performance index, radial displacement is applied to seFBG to generate the central wavelength shift of the seFBG. The seFBG signal is also generated as shown in Figure 10. After optimizing the interrogation method working in an approximately linear and most sensitive mode, the nano stage is used to conduct and feedback micro displacements. The nano stage is moved forward with a step displacement in the contact status of the twin FBG probe to find the minimum distinguished change. According to the experimental results shown in Figure 13, the wavelength resolution of the proposed interrogation method is 0.3 pm.

#### 3.2.5. Experiment on the Frequency Response

**Figure 14:**
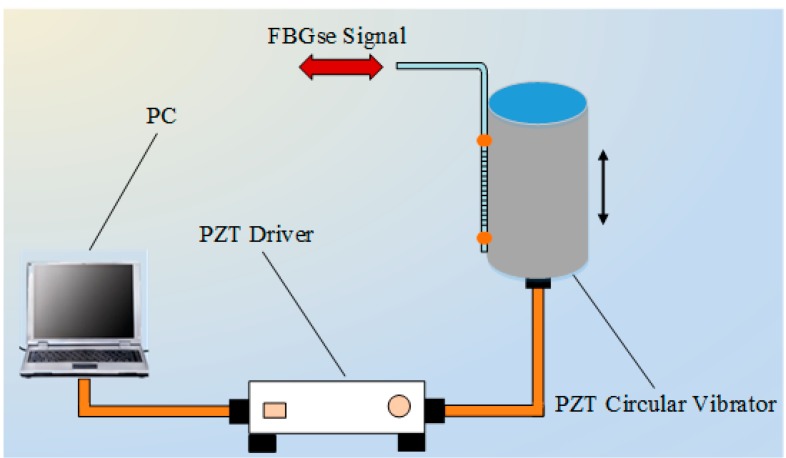
Schematic diagram of seFBG vibration signal generator.

**Figure 15:**
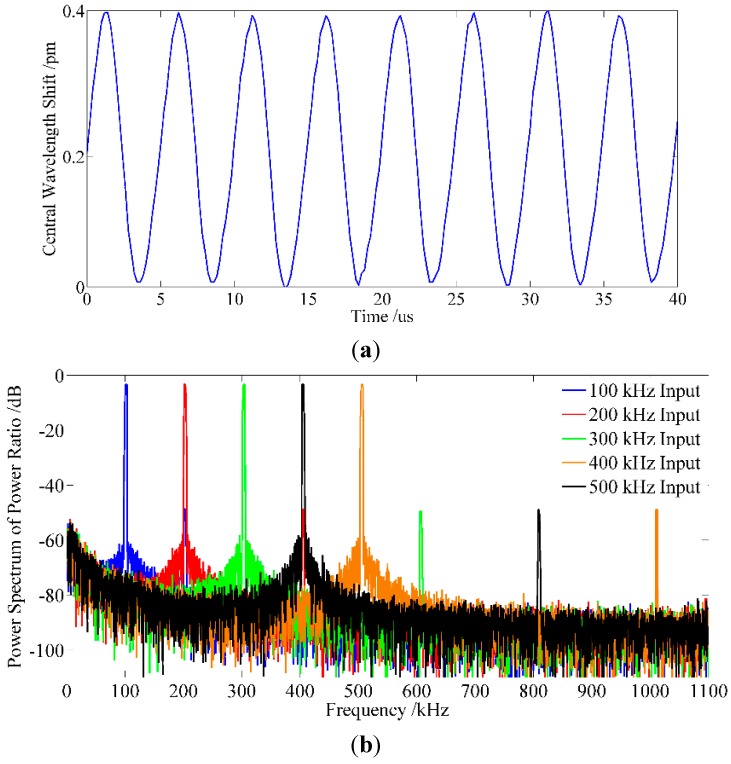
Experimental result of the frequency response: (**a**) temporal response resolution at 200 kHz; (**b**) high frequency responses at different frequency.

An experiment is conducted to verify the feasibility of the proposed method when applied to high speed interrogation. The seFBG is glued on a PZT circular vibrator along the direction of the thickness, and the location of the FBG is at the center of the wall as shown in Figure 14. The PZT circular vibrator applies a dynamic strain to the seFBG to simulate vibrations while it is driven by a sinusoidal signal at 100, 200, 300, 400 and 500 kHz, respectively, in multi-group experiments. The temporal responses of the system are firstly tested using a vibration of 200 kHz with a known peak-to-peak strain as shown in Figure 15a, and this interrogation method can clearly detect the ultrasonic waves and the peak-to-peak central wavelength shift is about 0.4 pm. Then, based on the Fourier transform analysis of power ratio sampling values, the frequency of power ratio can be obtained as shown in Figure 15b. The result indicates that the proposed interrogation can detect the vibration over a wide frequency range, even at 500 kHz, and higher frequency vibrations can be detected by using a high-performance DSP or other signal acquisition devices.

#### 3.2.6. Experiment on the Anti-temperature Perturbation Performance of the Proposed Interrogation Method

Temperature drift of the environment is the main factor influencing the measurement accuracy of FBG sensors. To verify the anti-temperature perturbation performance of the proposed interrogation method, an experiment on the proposed interrogation method under temperature perturbation is conducted.

**Figure 16:**
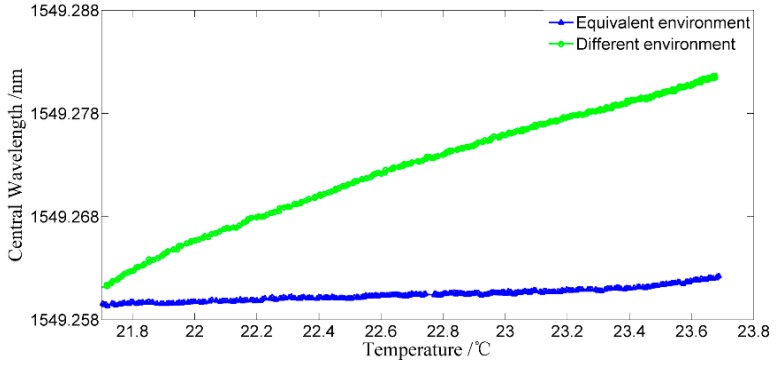
Experimental results of the anti-temperature perturbation performance of the interrogation method.

To compare the anti-temperature perturbation performance of the proposed interrogation method and other interrogation methods without a temperature compensation method, the seFBG and reFBG are firstly placed in the different environment (the other interrogation methods), and then the seFBG and reFBG are placed closely and exposed in an equivalent environment (proposed interrogation method). Figure 16 shows the central wavelength variations after the interrogation under two conditions, and it indicates that the influence of temperature on the proposed interrogation method has been significantly reduced to 9.5%, which approximately corresponds with the result of 6.04% calculated above.

## 4. Conclusions

A FBG interrogation method based on a reflective-matched FBG scheme is investigated in this paper. Aiming at addressing the nonlinear problem of this interrogation scheme, the optimal initial matching conditions are established to optimize the interrogation method working in its linear and most sensitive mode and this optimal initial matching condition can be achieved through a mechanical adjustment. The influence of the temperature drift given by theoretical analysis can be reduced to 6.04% which is an advantage of the proposed interrogation method. Before the performance test of the interrogation method, a well-designed acquisition circuit board is prepared to satisfy the measuring requirements of optical and electric signal processing and it achieves an optical power resolution of better than 8 pW; the mechanical adjustment is experimentally investigated, and the results show that the processes of compacting the reFBG without coating on the adjustment, twining reFBG around the cylindrical stage and immersing reFBG into an oil groove are beneficial to achieve a best power ratio stability of 0.06% with low stress relief. Experimental performance test results indicate that the nonlinearity is 3.3% in the measurable range of 60 pm, and the influence of temperature is significantly reduced to 9.5%, and the wavelength resolution and response speed can reach 0.3 pm and 500 kHz, respectively.

It can be concluded that the proposed interrogation method not only has the advantages of linear response and anti-temperature perturbation, but also has advantages of high response speed and high resolution. These properties suggest wide applicability to meet the high speed and high resolution requirements of FBG sensing systems.
